# Quantitative and qualitative changes in substance-related administrative offences in road traffic during the SARS-CoV-2 pandemic in Munich

**DOI:** 10.1371/journal.pone.0334598

**Published:** 2025-10-21

**Authors:** Anna Holzer, Andreas Stoever, Michael Lau, Sabine Gleich, Matthias Graw, Anouk Ludwig, Benno Hartung

**Affiliations:** 1 Institute of Legal Medicine, University Hospital Essen, Essen, Germany; 2 Institute of Legal Medicine, Ludwig Maximilians University Munich, Munich, Germany; 3 Mathematical Institute, Heinrich Heine University Düsseldorf, Düsseldorf, Germany; 4 Department for Health, City of Munich, Munich, Germany; University of Padova: Universita degli Studi di Padova, ITALY

## Abstract

**Introduction:**

The SARS-CoV-2 pandemic beginning in 2020 led to significant restrictions on social life and mobility, raising concerns about increased substance use across the general population. To investigate whether the pandemic resulted in quantitative or qualitative changes in alcohol and/or drug use in the context of road traffic, a retrospective analysis of toxicological findings was conducted in the city of Munich, considering the local pandemic-related restrictions.

**Materials and methods:**

A total of 6,210 blood samples were analyzed from individuals suspected of committing substance-related administrative traffic offences under §24a of the German Road Traffic Act between January 1, 2019, and July 31, 2021. Samples were examined for the presence of substances, their concentrations, and the type of vehicle involved. The cohort was stratified into pre-pandemic and pandemic periods, with March 16, 2020 set as the cut-off date. The pandemic period was further subdivided based on the severity of imposed restrictions. Statistical comparisons were performed using Fisher’s exact test, t-tests, ANOVA, and logistic regression.

**Results:**

Cannabis was the most frequently detected substance (66.2% pre-pandemic; 67.4% during the pandemic), followed by alcohol (11.7% vs. 10.8%) and cocaine (5.7% vs. 5.2%). Only minor differences were observed between the pre-pandemic and pandemic periods, as well as across phases of mild versus severe restrictions. Notably, THC-COOH concentrations were higher during the pandemic. Alcohol levels were elevated during phases of light restrictions and reduced during periods of strict lockdown. Cannabis was most commonly detected in car drivers, whereas alcohol was more frequently found in e-scooter riders, particularly during less restrictive phases.

**Conclusion:**

Substance detection patterns among drivers in Munich showed overall stability during the COVID-19 pandemic, with cannabis remaining the most commonly identified drug. However, shifts in substance concentrations and differences by vehicle type and restriction severity suggest subtle changes in consumption behavior. These findings underscore the need for continued surveillance and context-specific traffic safety measures.

## Introduction

Driving under the influence (DUI) remains a major global contributor to road traffic injuries and fatalities [1]. The COVID-19 pandemic significantly affected substance-related driving patterns worldwide. Lockdowns and reduced mobility resulted in lower traffic volumes; however, alcohol and drug use among drivers may have increased due to heightened stress, social isolation, and disrupted daily routines. Several studies reported elevated rates of alcohol and drug detection in seriously and fatally injured road users during the pandemic compared to pre-pandemic periods [2].

In Germany, DUI continues to pose a substantial road safety concern. In 2019, alcohol was reported as a contributing factor in approximately 4.6% of all traffic crashes resulting in physical injury, rising to 4.9% in 2020 and 5.3% in 2021. The rate of fatal crashes involving alcohol initially decreased to 5.7% in 2020, followed by an increase to 6.4% in 2021 [3]. Although one early study suggested a rise in impaired driving during the pandemic [4], subsequent research yielded more mixed results. Hostiuc et al. [5], for example, found no significant changes in blood or breath alcohol concentrations in Romanian drivers. Similarly, Marrone et al. [6] reported no notable shifts in the prevalence of alcohol or drug use among Italian drivers, although average blood alcohol levels were significantly higher during the pandemic period.

In Germany, operating a motor vehicle with a breath alcohol concentration (BrAC) of ≥0.25 mg/L or a blood alcohol concentration (BAC) of ≥0.5 g/kg constitutes an administrative offence under §24a of the German Road Traffic Act. A criminal offence may be considered if impairment is observed at a BAC of ≥0.3 g/kg, and any driver with a BAC ≥ 1.1 g/kg is deemed criminally liable regardless of observable impairment.

In addition to alcohol, driving a motor vehicle under the influence of cannabis, cocaine, amphetamine, methamphetamine or morphine – without showing any impairment – is also classified as an administrative offence according to §24a. In contrast, impaired driving under the influence of any central nervous system-active substance constitutes a criminal offence. For substances other than alcohol criminal liability requires evidence of impairment as no thresholds apply for criminal charges.

At the onset of the SARS-CoV-2 pandemic, it was hypothesized that not only individuals with substance use disorders but also vulnerable or high-risk individuals might increase their substance use [7, 8]. Self-reported data on alcohol and drug use during the pandemic have yielded mixed findings. While some studies reported no significant changes [9], others observed decreases [10–13], and still others indicated increases, particularly among individuals with preexisting substance use problems [10,13–16]. Using ethyl glucuronide (EtG) as a biomarker in hair samples, Alladio et al. [17] found a reduction in alcohol use among social drinkers during the initial lockdown in 2020, alongside an increase in chronic or heavy drinking as the lockdown progressed.

Given the heterogeneous findings on substance use during the pandemic, the aim of this study was to assess changes in administrative traffic offences related to alcohol and drugs among drivers in the Munich metropolitan area (population ~1.5 million) in relation to pandemic-related restrictions.

## Materials and methods

### Study design

This retrospective study analyzed toxicological reports from drivers suspected of substance-related administrative traffic offences under §24a of the German Road Traffic Act. The dataset comprised all cases involving blood sample collection for suspected substance use between January 1, 2019, and July 31, 2021, in the Munich metropolitan area.

Prior to blood sampling, individuals typically underwent roadside testing, including breath alcohol testing or urine drug screening, based on observed behavior or random testing. The decision to collect blood was made by law enforcement authorities. Collecting blood is always necessary if a drug use (other than alcohol) and/or a criminal offence is suspected. This study included only cases suspected of constituting administrative offences under §24a of the German Road Traffic Act.

Data were retrieved between January and December 2022. Relevant variables included age, sex (male, female, non-binary), vehicle type, sample collection date and time, and blood/breath test results.

The study was approved by the Ethics Committee of Ludwig Maximilians University of Munich (Reference: 21–0864).

### Alcohol and drug testing procedures

BAC was determined using gas chromatography and the alcohol dehydrogenase method [18]. Analyses were performed at the Institute of Legal Medicine in Munich and the Bavarian State Office for Health and Food Safety. BrACs were measured at police stations using Draeger Alcotest® 9510.

BAC results were reported in g/kg, while BrAC values (in mg/L) were converted to g/kg using a factor of 2.0 in accordance with §24a.

Drug screening was performed via immunoassay; positive samples were confirmed and quantified by liquid chromatography-tandem mass spectrometry (LC-MS/MS).

The following legal thresholds applied during the study period for administrative offences under §24a: THC: 1 ng/ml; benzoylecgonine: 75 ng/ml; cocaine: 10 ng/ml; morphine: 10 ng/ml; amphetamine, MDMA, MDE, MDA, methamphetamine: 25 ng/ml.

### Study population and sub-cohorts

**Fig 1:**
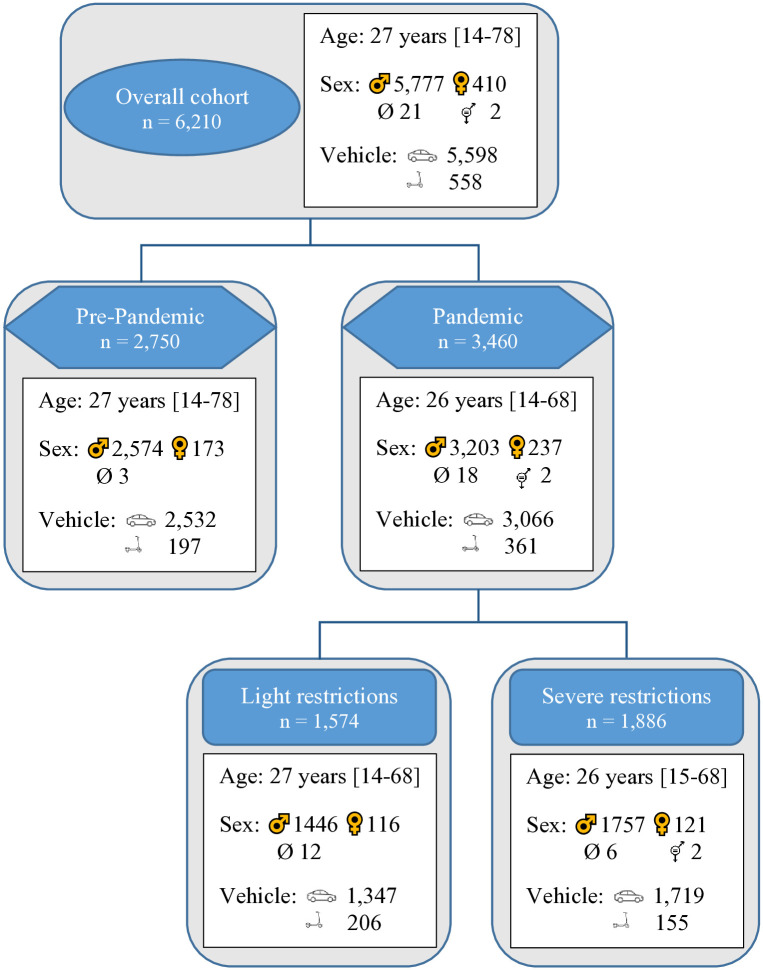
Study population stratified by pandemic periods, demographics, and the most common vehicle used. The study population is comprised of individuals who were suspected of an administrative traffic offence related to substance use in Munich between January 1, 2019 and July 31, 2021.

A total of 6,210 individuals were included in the final analysis. Participants were categorized into the following sub-cohorts (Fig 1):

#### Pre-pandemic vs. Pandemic periods.

Two temporal cohorts were defined: a **pre-pandemic** cohort (January 1, 2019 – March 15, 2020) and a **pandemic** cohort (March 16, 2020 – July 31, 2021). The cut-off date corresponds to the declaration of a state of emergency in Bavaria, which led to widespread closures of public institutions and events [19].

#### Impact of restriction severity.

Pandemic-related public health measures were categorized into three levels of restriction severity based on data from Munich’s Department of Health (see S1 Table).

- **No restrictions:** January 1, 2019 – March 15, 2020.
- **Light restrictions:** Periods with partial reopening of restaurants and cultural venues or the temporary suspension of emergency status: May 13 – September 23, 2020; March 8 – April 3, 2021; and May 30 – July 31, 2021.
- **Severe restrictions:** Periods with lockdowns, curfews, and the closure of public venues: March 16 – May 12, 2020; September 24, 2020 – March 7, 2021; and April 4 – May 29, 2021.

#### Seasonal effects (warm and cold season, Oktoberfest).

To examine the influence of seasonal variation, the calendar year was divided into a warm and cold season. Additionally, Oktoberfest — an event known for its alcohol-related impact – was analyzed separately to isolate its potential effect.

The **warm season** was defined as April 16 – October 15, 2019, and the **cold season** as January 1 – April 15, 2019 and October 16, 2019 – March 15, 2020. These cut-off dates are based on average air temperatures in Germany between 2001 and 2020, as reported by the German Weather Service [20]. April and October midpoints were chosen to represent a balanced six-month period with similar temperatures at both ends. The 2019 Oktoberfest took place from September 21 to October 6 and was evaluated independently. As Oktoberfest was cancelled in 2020 and 2021, only pre-pandemic data were included in the seasonal analysis.

### Statistical analysis

Standard descriptive statistical tests were employed to analyze the data.

For categorical comparisons between pre-pandemic and pandemic periods, as well as across different levels of pandemic restrictions (e.g., vehicle type and substance frequencies) Fisher’s exact test was applied.

For continuous variables (e.g., substance concentrations), two-sample t-tests were used to compare between the pre-pandemic and pandemic periods. One-way analysis of variance (ANOVA) was employed to compare concentrations across the different levels of restriction severity.

Associations between vehicle type and substance detection were examined using logistic regression and ANOVA.

Seasonal effects were analyzed by Fisher’s exact test. To isolate the effect of Oktoberfest from general seasonal variation, a multiple logistic regression model was used, including season and event variables.

All analyses were conducted in R.

## Results

### Pre-pandemic vs. Pandemic periods

A total of 6,210 blood samples were analyzed, with 2,750 samples classified as pre-pandemic and 3,460 as pandemic cases. The two groups were comparable in age and sex distribution (Fig 1). Although the daily average of substance-related traffic offences was marginally higher during the pandemic (6.79/day vs. 6.25/day), this difference did not reach statistical significance (p = 0.06).

There was a statistically significant change in the types of vehicles involved. Car-related offences declined from 92.1% to 88.6%, while e-scooter-related offences increased from 7.2% to 10.4% during the pandemic (p < 0.01).

Cannabis was the most frequently detected substance both pre-pandemic (66.2%) and during the pandemic (67.4%), followed by alcohol and cocaine. The proportion of multi-substance cases was slightly lower during the pandemic (13.7% vs. 14.8%), and approximately 11% of all samples contained no detectable substance in both periods.

**Fig 2:**
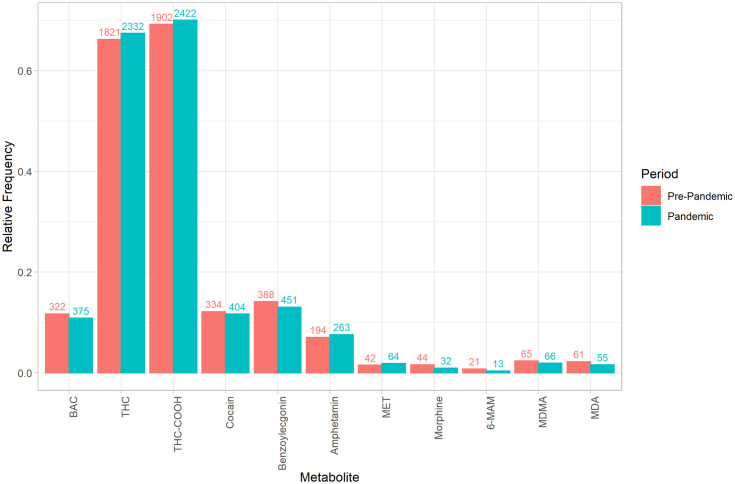
Frequency of substances and its metabolites detected in the blood samples from individuals suspected of having committed an administrative traffic offence in Munich, stratified by pre-pandemic and pandemic periods. The y-axis denotes the relative frequency, while the labeling of the bars indicates the absolute frequency. Statistical differences between pre-pandemic and pandemic periods were assessed using Fisher’s exact test. Statistically significant differences were identified for morphine (p < 0.05).

A significant reduction was observed for morphine detection during the pandemic (1.6% vs. 0.9%, p < 0.05) (Fig 2).

**Fig 3:**
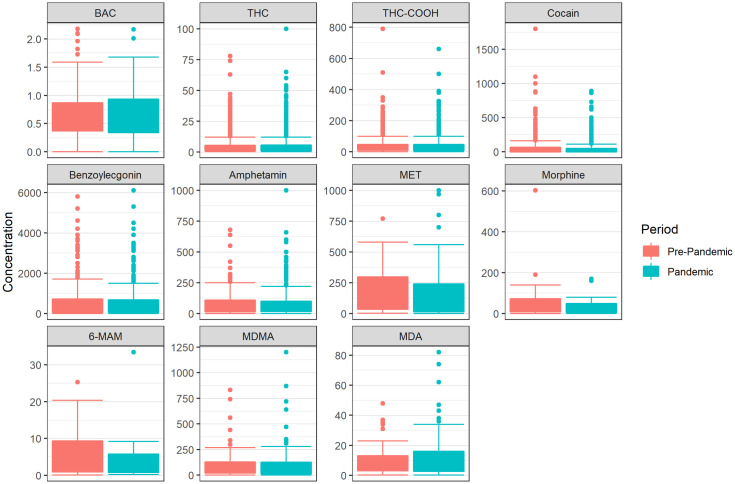
Substance concentrations in blood samples from individuals suspected of having committed an administrative traffic offence in Munich, stratified by pre-pandemic and pandemic periods. The BAC is indicated in g/kg. The concentrations of THC, THC-COOH, cocaine, benzoylecgonine, amphetamine, methamphetamine, morphine, 6-monoacetylmorphine (6-MAM), MDMA and MDA are indicated in ng/ml. Statistical differences between pre-pandemic and pandemic periods were assessed using two sample t-tests per substance. Statistically significant differences were identified for THC-COOH (p < 0.05).

Substance concentrations remained largely stable, except for THC-COOH, which was significantly higher during the pandemic (p < 0.05). Mean BACs (0.62 g/kg) were unchanged in both periods (Fig 3).

After excluding samples below or above the legal thresholds defined by § 24a (resulting in the exclusion of 22% of cases), the pattern remained similar. In this threshold-adjusted cohort, cocaine overtook alcohol as the second most frequently detected substance after cannabis.

**Fig 4:**
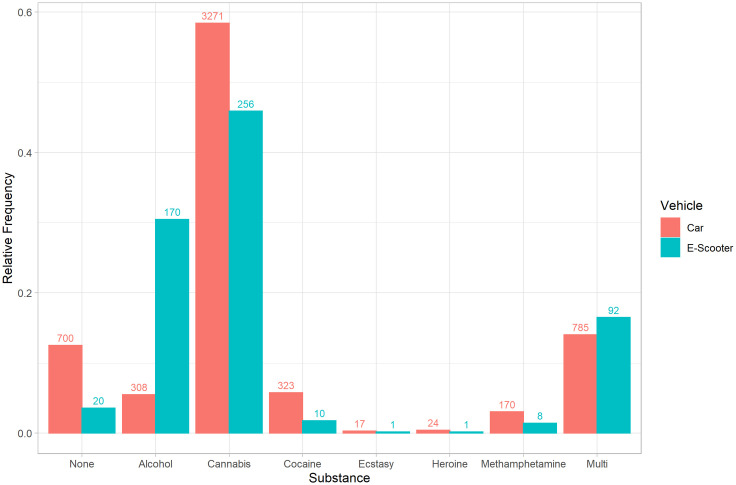
Frequency of substances detected in the blood samples from individuals suspected of having committed an administrative traffic offence in Munich, stratified by car drivers (n = 5,598) and e-scooter riders (n = 558). The y-axis denotes the relative frequency, while the labeling of the bars indicates the absolute frequency. Blood samples containing multiple substances are designated as “multi” in the figure. Statistical associations between observed vehicle type and detected substance were assessed using logistic regression with ANOVA. Statistically, e-scooter riders are more prone to alcohol-related offences, while car drivers are more susceptible to cannabis-related offences (p < 0.01).

In regards of vehicle-specific associations, e-scooter riders were more often positive for alcohol, while car drivers more frequently tested positive for cannabis (p < 0.01). Multi-substance use was also slightly more common among e-scooter riders (Fig 4).

### Impact of restriction severity

**Fig 5:**
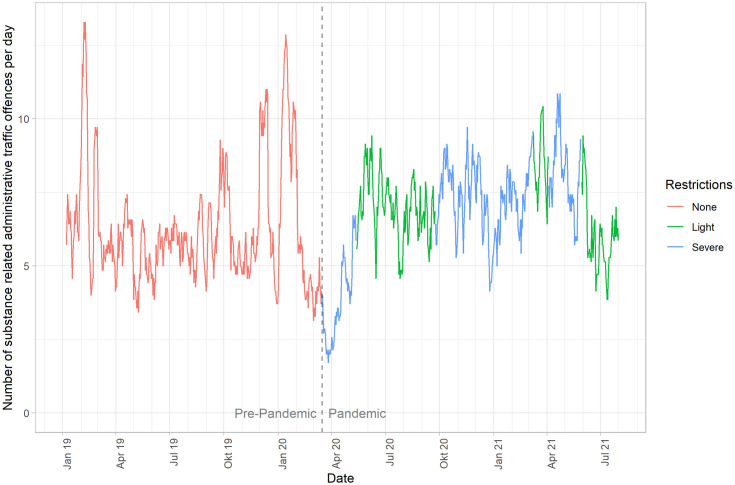
Number of individuals per day assigned for blood sampling due to suspicion of committing an administrative traffic offence in Munich, stratified by periods of varying pandemic restriction levels. Statistical differences were assessed using Fisher’s exact test. Statistically significant differences were not identified (p = 0.08).

The sample was further divided into no restrictions (n = 2,750), light restrictions (n = 1,574), and severe restrictions (n = 1,886). Demographic characteristics were comparable across these groups (Fig 1). The number of offences temporarily declined during the initial lockdown, but no sustained trend was evident over time (p = 0.08) (Fig 5).

Vehicle types varied across restriction severity, as car-related offences declined during light restriction phases, while e-scooter-related offences increased (p < 0.01).

**Fig 6:**
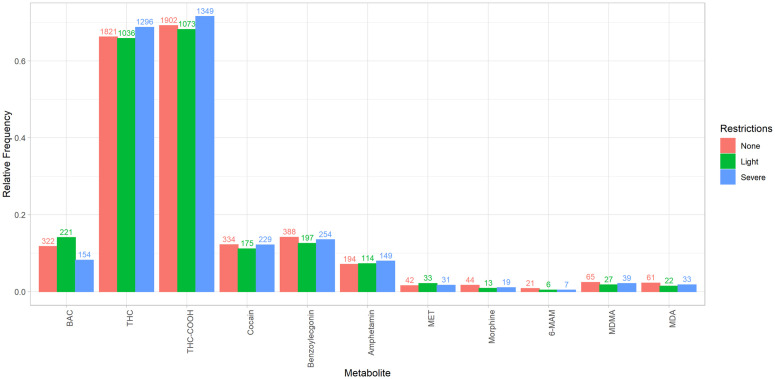
Frequency of substances and its metabolites detected in the blood samples from individuals suspected of having committed an administrative traffic offence in Munich, stratified by periods of varying pandemic restriction levels. The y-axis denotes the relative frequency, while the labeling of the bars indicates the absolute frequency. Statistical differences between pandemic restriction levels were assessed using Fisher’s exact test. Statistically significant differences were identified for alcohol (p < 0.01) and cannabis (p < 0.05).

Substance detection patterns remained mostly stable, except for alcohol and cannabis: alcohol was less frequently detected during severe restrictions (p < 0.01), while cannabis was more commonly detected during those same periods (p < 0.05) (Fig 6).

**Fig 7:**
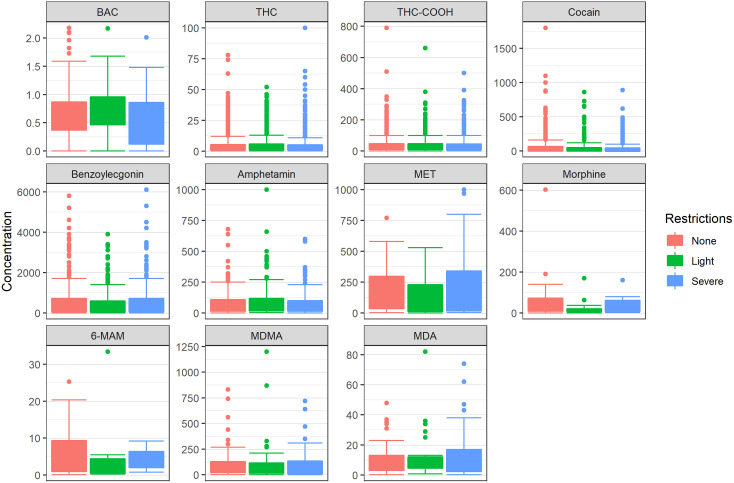
Substance concentrations in blood samples from individuals suspected of having committed an administrative traffic offence in Munich, stratified by periods of varying pandemic restriction levels. BAC is indicated in g/kg. The concentrations of THC, THC-COOH, cocaine, benzoylecgonine, amphetamine, methamphetamine, morphine, 6-MAM, MDMA and MDA are indicated in ng/ml. Statistical differences between pandemic restriction levels were assessed using ANOVA. Statistically significant differences were identified for alcohol (p < 0.01) and THC-COOH (p < 0.05).

Substance concentrations reflected these changes accordingly: alcohol levels were significantly higher during light restrictions compared to both no and severe restrictions (p < 0.01), while THC-COOH concentrations raised during severe restrictions (p < 0.05) (Fig 7).

After adjusting for legal thresholds, alcohol-related offences showed similar significant findings (p < 0.01).

### Seasonal effects

Alcohol-related offences were significantly more frequent during the warm season (12.5%) than during the cold season (5.7%, p < 0.01). Notably, 23.9% of all alcohol detections in the warm season occurred during Oktoberfest (p < 0.01).

Cannabis detection decreased slightly during Oktoberfest (p < 0.05), while no significant seasonal variation was observed for other substances. Interestingly, the proportion of substance-negative samples was significantly higher during the cold season (p < 0.01).

## Discussion

This study aimed to evaluate the impact of the COVID-19 pandemic on alcohol and drug use among individuals suspected of substance-related administrative traffic offences in Munich. By analyzing toxicological data before and during the pandemic, and across varying levels of restriction severity, we found that substance detection patterns remained broadly consistent. However, some notable context-specific shifts were observed.

Cannabis remained the most commonly detected substance, particularly among car drivers. Although overall detection rates were stable, the significant increase in THC-COOH concentrations suggests more frequent or more intensive cannabis use during the pandemic. Since THC-COOH is an inactive metabolite, its elevated levels may indicate regular or chronic consumption. This aligns with national survey data indicating an increase in daily or near-daily cannabis use in Germany during the pandemic [21, 22].

Alcohol detection showed a more dynamic pattern. While overall detection rates did not differ significantly, alcohol was more frequently detected during periods of light restrictions and in the warm season. Detections decreased during strict lockdowns, consistent with reduced mobility and social activity during those phases.

The seasonal variation – particularly the spike during Oktoberfest and the rise of substance-negative samples in winter – reflects known temporal patterns in alcohol-related offences and confirms the relevance of operational factors as well as cultural and social events in influencing behavior [23–25]. This point warrants further investigation.

E-scooter users were more frequently positive for alcohol than car drivers, particularly during phases of light restriction. This suggests a possible shift in micro-mobility patterns, where alcohol-impaired individuals may have opted for e-scooters. Our findings differ from Eppler et al. [23], who observed declining e-scooter-related offences post-2019. This discrepancy may stem from methodological differences (e.g., inclusion/exclusion of BrAC-only cases) or local enforcement intensity.

Contrary to early hypotheses (e.g., Beccegato et al. [4]), our data do not support an overall increase in impaired driving during the pandemic. While the number of collected samples slightly increased during the pandemic, this does not necessarily reflect a true rise in impaired driving incidents. Munich’s population remained relatively stable during the study period, and according to police authorities in Munich, no structural changes in control practices were implemented during the pandemic. Additionally, cellphone-based mobility data showed a significant decrease in movement during lockdowns [26, 27]. Therefore, the increase may be attributable to steady enforcement despite temporarily reduced traffic volume. This finding is consistent with Hostuic et al. [5] and others [6, 28], suggesting that while substance use behavior may have shifted in private settings, this was not reflected in a rise in substance-related offences in traffic.

Our data do not directly support a general increase in car usage during the pandemic [29], but they suggest a relative shift in substance-related offences toward e-scooter riders. These trends could reflect a change in preferred modes of transport during a pandemic.

Although we did not perform statistical subgroup analyses by age or gender, we acknowledge that both factors may influence the patterns observed, especially considering the overrepresentation of young males among cannabis users and e-scooter riders.

### Limitations

This study is limited by its geographic scope, as it reflects data from a single urban area in Germany and may not be generalizable to other cities, rural regions, or national trends. Pandemic-related restrictions varied across German states, with Bavaria often enforcing stricter measures than other regions.

Selection bias cannot be excluded, as traffic controls were not only performed randomly and may have been influenced by law enforcement priorities, particularly during periods of public health concern or major events such as Oktoberfest [23] or during strict lockdown phases when social interaction was heavily restricted. The absence of precise traffic volume data further limits the ability to interpret changes in detection rates. A decline in driving volume, especially during lockdowns, may have artificially reduced the number of offences without reflecting actual changes in driver behavior.

Finally, the analysis did not include a more detailed stratification by age, sex, or other demographic characteristics. Although we report the overall demographics of the sample, future analyses may help clarify how individual characteristics affect substance use patterns in traffic contexts.

## Conclusion

This study presents a comprehensive analysis of alcohol and drug use among individuals suspected of substance-related administrative traffic offences in Munich during the COVID-19 pandemic. While the overall detection rate of most substances remained stable across the study period, several nuanced changes emerged. Cannabis consistently emerged as the most frequently detected substance, particularly among car drivers. Elevated levels of THC-COOH during the pandemic suggest an increase in frequent or habitual use. Alcohol-related offences fluctuated with public health restrictions, showing higher detection rates during relaxed phases and lower rates during strict lockdown. E-scooter riders were more often involved in alcohol-related offences, highlighting a potential area for targeted traffic safety policies. Seasonal factors, such as Oktoberfest and summer months, were associated with increases in alcohol detections, while winter months saw a higher proportion of substance-negative samples.

These findings underline the complex interplay between public health measures, individual behavior, and enforcement practices. They emphasize the need for adaptive prevention strategies and continuous monitoring of mobility trends, particularly regarding micro-mobility and its potential risks.

Future research should incorporate traffic volume data, explore demographic subgroups, and expand to include additional geographic regions to support the generalizability of these results.

## Supporting information

S1 Table Chronological list of the pandemic measures enacted in Munich due to the SARS-CoV-2 pandemic. (PDF)
