# Supplementary material for: Quantitative and qualitative changes in substance-related administrative offences in road traffic during the SARS-CoV-2 pandemic in Munich
Source: PLoS One. 2025 Oct 21;20(10):e0334598. doi: 10.1371/journal.pone.0334598 (PMC12539698; doi:10.1371/journal.pone.0334598)
Supplement: S1 Table — (PDF) [file pone.0334598.s001.pdf]

Date	Measurement	
27.01.2020	A patient infected with the **novel coronavirus** (SARS-CoV-2) is admitted to the hospital Klinikum Schwabing in Munich. This is the first patient in Germany with this diagnosis.	
28.02.2020	Due to the spread of coronavirus infections in northern Italy and new cases in Germany, Munich's first mayor Dieter Reiter appoints a **Staff for Extraordinary Events** (SAE) in order to quickly initiate necessary measures.	
02.03.2020	The city sets up a **citizens' hotline** for the first time to answer questions about the coronavirus. City employees staff the hotline service daily from 8 am to 6 pm all year round - even in summer, when the infection situation eases.	
05.03.2020	Munich **schools and child daycare centers** are closed for the first time due to confirmed coronavirus infections.	
11.03.2020	The City of Munich launches a **coronavirus testing station on the site of the former Bayernkaserne barracks,** intended for individuals who have had contact with a person infected with the coronavirus and have thus become a 1st degree contact person. Drive-in testing is carried out in cooperation with the Aicher Group GmbH & Co. KG. Starting March 26, the City of Munich will create additional testing capacities in the Bayernkaserne for individuals who are employed in critical infrastructure facilities, in retirement and nursing homes, and in the medical sector.	
11.03.2020	Mayor Dieter Reiter orders the **closure of the city's theaters** due to the coronavirus pandemic. By April 19, all theaters and concert halls like the Gasteig and the Pasinger Fabrik must shut down their operations. The following day, the State of Bavaria bans all events with more than 1.000 people. On March 14, the municipal museums, art spaces, the NS Documentation Center, the Munich City Library and the Munich Adult Education Center have to close by order of the government of Bavaria.	
16.03.2020	With the declaration of a **state of emergency for Bavaria** due to the coronavirus pandemic, all public events in Munich are prohibited and all leisure facilities, including public swimming pools, are closed. Retail stores are also affected by the closures - with the exception of stores that provide daily supplies. Restaurants have to close as well. The **municipal offices** reduce personal contact to a minimum and switch to contacting citizens by email and telephone. In addition, **all schools, kindergardens and childcare centers** are closed. Despite the closures, emergency childcare is still available for employees in essential areas. Citizens in acute emergencies continue to receive in-person support in social community centers or are visited at home.	
16.03.2020	In cooperation with the Ambulatory Health Association Bavaria and Ambulanz-Rettung und Zivilschutz gGmbH, the City of Munich establishes another **drive-in testing station for coronavirus** at the **Theresienwiese**. This testing site is intended exclusively for individuals who have a reasonable suspicion of a coronavirus infection based on a medical history taken by telephone.	
17.03.2020	The UEFA Executive Committee decides to postpone the **2020 European Football Championship** to 2021 due to the coronavirus pandemic.	
18.03.2020	The plenary session of the City Council decides on **immediate economic measures** to prevent the insolvency of municipal companies and institutes or to protect municipal grant recipients in view of the drastic measures to combat the coronavirus pandemic. It is decided that the City of Munich will **support sporting events and projects of sports clubs**, even if they have been postponed or canceled.	
20.03.2020	The city of Munich sets up **central contact points for volunteers**. Not only medical staff such as doctors, nurses or students from medical faculties are sought, but also volunteers who would like to offer the elderly help with shopping or important errands, for example.	
21.03.2020	With the **lockdown** taking effect throughout Bavaria, public life in Munich winds down significantly. All Munich residents are required to reduce personal contacts to an absolute minimum. Wherever possible, a minimum distance of 1.5 meters between individuals must be maintained. All restaurants, hairdressers, florists and any retail stores that do not urgently serve daily needs will generally remain closed. Supermarkets, grocery stores, drugstores, petrol stations and banks remain open.	

	Restaurants that offer or deliver take-away food may stay open. Going to work, shopping for groceries, to the pharmacy and urgent visits to the doctor are still permitted. Outdoor sports may also be practiced - but only alone or with immediate family.	
21.03.2020	The Social Services Department enables homeless persons to stay in the **overnight shelter in the Bayernkaserne** for the entire day - including meals. The city council extends the measure until March 31, 2020 due to the pandemic.	
23.03.2020	The **fire department's danger prevention management** takes up its work. It is responsible, for example, for the city's logistics for protective equipment and disinfectants as well as coordinating the distribution of Covid-19 patients to Munich's hospitals.	
23.03.2020	The Social Services Department now offers a **service hotline** as a primary point of information for citizens. It connects individuals to one of the twelve social community centers or to the Munich Jobcenter, as well as to the Office for Housing and Migration for questions about homelessness and insecure housing situations. In addition, the Social Services Department launches a **shopping service** for elderly persons at all twelve social community centers and the 32 retirement and service centers.	
24.03.2020	The first **Corona Emergency Aid Payments** are transferred to small and medium-sized companies in Munich. Companies and freelancers with up to 250 employees may apply for this financial aid from the State of Bavaria if they experience liquidity difficulties due to the coronavirus crisis. The Department of Labor and Economic Affairs is responsible for reviewing the companies' applications.	
27.03.2020	The City sets up a **donation account** to support social organizations and projects in Munich. The aim is to provide quick and targeted help in acute emergencies. The donations also go into the "Help the helpers!" campaign launched by the City of Munich.	
30.03.2020	The sponsors of the station mission, Innere Mission and Caritas, together with the social welfare department, further expand the **services for those in need in the city center**. A food truck is put into operation on Karl-Stützel-Platz, followed by a second one in Schwanthalerstraße, where clothing for women, men and children are distributed as well.	
03.04.2020	Following the cancellation of the Auer May fair due to the coronavirus pandemic, the Department of Labor and Economic Affairs, in cooperation with the Bavarian State Association of Market Merchants and Showmen, launches the **"Fair is cult!" voucher campaign** to support the affected market merchants and showmen. Fair fans can buy vouchers and redeem them at one of the next Auer May fairs.	
08.04.2020	The City Council is presented with **immediate measures** planned by the municipal housing companies GEWOFAG and GWG to protect tenants affected by the economic impact of the coronavirus pandemic. For example, the deferment of owed rents is made possible in cases of hardship caused by the corona crisis.	
16.04.2020	**#muenchenhältzamm** - under this motto, the Department of Labor and Economic Affairs and the city portal muenchen.de create a web platform to support companies affected by the coronavirus crisis and ensure their continued existence.	
21.04.2020	The world's largest fair, the **Munich Oktoberfest**, is cancelled in 2020 due to the coronavirus pandemic. This was announced by Mayor Dieter Reiter and Bavaria's Minister-President Dr. Markus Söder.	
22.04.2020	The first wave of Covid-infections reaches its peak at **Munich's city hospitals**. Around 200 patients are treated simultaneously at four locations, 70 of them in the intensive care unit.	
25.04.2020	On behalf of the Bavarian State Department of Education, the fire department distributes **80.000 face masks** to schools in Munich with the support of the volunteer fire departments in Munich. The goal is to ensure that classes can resume.	
27.04.2020	The pupils of **graduating classes** are back in school. At the Munich public schools, including 14 grammar schools and 20 secondary schools, there are 160 graduating classes with 3.400 pupils. At the 83 public vocational schools, around 20.000 pupils attend final-year classes.	

04.05.2020	The inaugural meeting of the newly elected City Council takes place in the Deutsches Theater due to the coronavirus distancing rules. The following **plenary sessions of the City Council** with 80 city council members have to move due to hygiene regulations from the town hall to the Gasteig, Löwenbräukeller and Showpalast in Fröttmaning.	
04.05.2020	The city administration reopens to restricted **political party services**. As in stores and on public transport, the obligation to wear a face mask and the minimum distance requirement of 1.50 meters apply. The offices of the district administration department have used the forced break to expand digital services and optimize processes. As legally possible, services and consultations are now offered online, by email, by telephone and by mail.	
04.05.2020	The city develops a package of measures to quickly and efficiently support approximately **700 Munich sports clubs** during the coronavirus crisis. This includes distributing sports operating grants as well as subsidies for the maintenance of club-owned sports facilities to the clubs earlier than scheduled. The club lump sum from the State of Bavaria, which is paid by the city to the clubs, is also transferred sooner.	
06.05.2020	The **coronavirus measures are gradually eased**. The general lockdown is dropped. Contact restrictions and social distancing rules continue to apply. Classes in schools will gradually resume in the coming weeks, childcare will be gradually expanded, retail and service businesses will be able to reopen, followed by restaurants. The city's museums, the NS Documentation Center Munich, the Munich City Library, Hellabrunn Zoo and the city's outdoor sports facilities will also reopen to the public under certain conditions. Playgrounds in the city's green spaces are also reopened, soon followed by leisure and sports facilities that were forced to close on March 17.	
13.05.2020	The City Council decides to temporarily set up **additional outdoor restaurant areas**, especially in parking lots, to support the food industry, which has been severely affected by the coronavirus. In a very short time, the district inspectorates of the district administration department process more than 1.100 corresponding applications, thus enabling almost 9.000 additional outdoor seating units to compensate for the reduced number of seating areas due to the social distancing regulations. Munich's new "Schanigärten" have a lasting impact on the cityscape and are very well accepted.	
20.05.2020	The City Council decides that the city will not charge sports clubs for the use of indoor and other sports facilities during the coronavirus-related **closure of the city's sports facilities**.	
06.07.2020	Munich's sports clubs are able to practice in the city's school **sports facilities** again. The Department of Education and Sport has developed a protection and hygiene concept that makes it possible to use the city's sports facilities even in times of coronavirus. This is followed quickly by the reopening of the city's outdoor school sports facilities.	
30.07.2020	After a temporary closure, the **corona testing station on the Theresienwiese** is back in operation. The reason is the State's policy of offering low-threshold testing for everyone. For example, asymptomatic people who need a PCR test for travel purposes or who have received a notification via the Corona-Warn-App can get tested. Due to the high demand, testing capacities are constantly increased. In December, 2.500 tests per day are possible from Monday to Friday. Saturday and Sunday both offer 600 tests per day. This means that up to 13.700 tests per week can be performed at the Theresienwiese.	
01.09.2020	The 2020/21 preschool year starts in regular operation in compliance with the hygiene measures set by the State of Bavaria. Less than a week later, the new school year 2020/21 starts with a focus on the best possible protection against infections in schools.	
10.09.2020	The City of Munich issues a temporary **ban on the sale and consumption of alcohol** in public places in well-known hotspots on the weekends. The aim is to keep the coronavirus infection rate under control as much as possible. Taking into account the decisions of the Bavarian Administrative Court, the new general order is restricted to hotspots and only applies to the most problematic days of the week: Friday evening to Sunday morning.	

24.09.2020	The City of Munich has issued a **general mask wearing mandate** in parts of the city center every day from 9 am to 11 pm. This applies in the Old Town pedestrian zone including Sendlinger-Tor-Platz, at Rindermarkt, Viktualienmarkt, in Dienerstraße, Schrammerstraße and Landschaftstraße, in Schützenstraße, in the Stachus underground and on the sidewalks in the Tal.	
24.09.2020	In addition, **spending time together in private and public places** and at a shared table in restaurants is limited to families and partners or groups of up to 5 people. Private parties, club and party meetings as well as non-public events and gatherings are generally only permitted for up to 25 participants in closed rooms or up to 50 participants in the open air if the organizer has drawn up a protection and hygiene concept and can present it on request.	
28.09.2020	The City of Munich receives **administrative assistance** from the German Armed Forces in identifying and supporting category 1 (KP1) coronavirus contacts. More than 50 soldiers support the contact tracing teams (CTT) of the public health department.	
17.10.2020	In view of the rapidly increasing number of coronavirus infections, the **coronavirus measures of the State of Bavaria are intensified**. There is a general requirement to wear masks in the Old Town pedestrian zone, on the sidewalks in the Tal as well as in Schützenstraße and in the Stachus underground. Masks must also be worn in public places, including elevators in public buildings, leisure facilities and cultural venues. In addition, masks are now mandatory in theaters, concert halls, other stages and cinemas, at conferences and congresses and for visitors at sporting events, even when seated. Spending time together in private and public spaces and at a shared table in restaurants is only permitted in groups of up to 5 people or two households. A curfew applies after 10 p.m. in the restaurants, with the exception of takeaway food and non-alcoholic drinks. In addition, alcohol consumption in public places is prohibited daily from 10 p.m. until 6 a.m. at known hotspots. Alcohol may no longer be sold at petrol stations, other sales premises and delivery services after 10 pm.	
23.10.2020	The on-call clinic "Infekt" at the Theresienwiese in Munich is a **new medical service** for all patients with typical symptoms of flu or severe colds such as cough, sore throat and fever. The facility run by the Ambulatory Health Association Bavaria is an important addition to the existing outpatient care facilities in Munich.	
28.10.2020	The City of Munich **prepares** the **coronavirus testing station** at the Theresienwiese **for winter**. A large "Wiesn tent" is set up to protect both the tested persons and the medical staff from the cold, wind and rain.	
30.10.2020	The City of Munich cancels the **Christmas market** on Marienplatz due to the intensified pandemic. The Christmas markets in the city districts cannot take place either. The carnival kick-off on November 11, the dance of the market wenches 2021 and the citizens' meetings for the rest of 2020 are also canceled.	
02.11.2020	A **partial lockdown**, in which the federal and state governments have agreed on the same restrictions for public life, is intended to prevent an imminent overload of the healthcare system with severe Covid-19 cases. After just a few months, cultural and leisure facilities have to close again. This also affects museums, exhibitions, theaters and orchestral venues and the Olympic Park. Restaurants are only allowed to sell take-away food. Contact restrictions and alcohol bans are tightened. Hellabrunn Zoo, swimming pools and saunas have to close again. In contrast to the first lockdown, schools and childcare facilities remain open, as do retail outlets.	
09.11.2020	**München Klinik** takes stock: since the start of the pandemic, a total of 1.000 Covid-19 patients have been treated in the city's clinics. By the end of November, the number of patients, which had decreased temporarily, rose again, reaching 200 towards the end of the month, the same number as during the peak phase in spring.	
01.12.2020	The Health Department's **Contact Tracing Teams (CTT)** are centralized in a hall at the Munich Trade Fair in Riem. Optimal working conditions were created for around 500 employees, including staff from the Department of Health and Environment (RGU) and other departments of the city, soldiers from the German Armed Forces and supporting staff from the police, the State of Bavaria and the Robert Koch Institute.	

09.12.2020	Due to the consistently high number of infections, the Bavarian state government once again declares a **state of emergency**. In addition, stricter corona measures come into force with a Bavaria-wide **curfew**. The extended regulations for hotspots also apply to Munich, as the 7-day incidence value reported by the Robert Koch Institute (RKI) has exceeded the threshold value of 200. This means that in addition to the new curfew in Munich, there is also a night-time curfew between 9 pm and 5 am. Furthermore, **distance classes** are introduced at Munich schools (including business schools) for 8th-graders and higher until Christmas break. This excludes final-year classes and special schools. Classes at vocational schools will also switch to distance classes; at technical and vocational high schools (FOS/BOS), this applies to the preparatory class and the 11th grade. In addition, an **all-day ban on alcohol consumption** applies in public spaces throughout the city. The **mandatory wearing of masks** in public spaces remains unchanged.	
15.12.2020	The city has set up a **vaccination center at the trade fair grounds in Munich** so that vaccinations can start as soon as a coronavirus vaccine is available. In the first expansion stage of the vaccination center, up to 20 vaccination teams are available. They are formed by the Aicher Ambulanz Union in cooperation with MKT - Krankentransport OHG and will soon vaccinate staff and residents of retirement and nursing homes, predominantly as mobile teams.	
16.12.2020	A **nationwide lockdown** is implemented due to a rising number of infections. In addition to the coronavirus measures already in force in Munich, retailers (with the exception of food and everyday products) and hairdressers must initially close until January 10. Furthermore, schools and daycare centers will be closed except for emergency childcare. At Christmas, one household may meet with four other people from the closest family circle. A ban on the sale of pyrotechnics and a night-time curfew apply for New Year's Eve.	
27.12.2020	Right on time for the nationwide start of vaccination, the **first individuals** in Munich **get vaccinated** against the coronavirus. The vaccine is given to residents and employees of a nursing home in Freimann.	
15.01.2021	Following the nationwide kick-off of the vaccination campaign, the city of Munich sets up a **vaccination hotline** where Munich residents who do not have their own internet access may register for a coronavirus vaccination appointment at the Riem vaccination center.	
22.01.2021	The City of Munich has almost completed the distribution and dispatch of around 500.000 FFP2 face masks to Munich citizens. Due to the corona pandemic, citizens over the age of 15 have been **required to wear an FFP2 face mask** in shops and on public transportion throughout Bavaria since January 18.	
03.02.2021	The city's vaccination center at the Messe München trade fair center starts the regular **corona vaccinations**. The first 450 vaccination appointments are available until February 5. Initially, only individuals in the **highest priority group** are vaccinated, including the over-80-year-olds as well as medical and nursing staff. In total, around 120.000 people in Munich fall into this highest priority category.	
08.03.2021	With the new Bavarian Infection Protection Measures Regulation, **new corona rules** are implemented that make contact restrictions and the opening of retail outlets, museums, zoological gardens and sports facilities **dependent on the level of the 7-day incidence value**. Because the incidence in Munich is between 50 and 100, private gatherings of two households with a maximum of five people are possible. Retailers may offer appointment shopping. City museums, Munich City Library facilities and Hellabrunn Zoo may reopen in compliance with coronavirus regulations.	
16.03.2021	The first two **GP offices** in Munich start **offering coronavirus vaccinations**. The aim is to test the processes in the GP offices and the handling of the new vaccines. The following day, the first local vaccinations start in retirement and service centers, the ASZ Untergiesing being the first. Vaccination campaigns will also be initiated for the homeless and for refugees.	

26.03.2021	The traditional **Auer May Fair** has to be **canceled** again this year due to the pandemic. This decision was made by the staff for extraordinary events (SAE) due to the dynamic infection rate with the spread of the coronavirus variant B.1.1.7.	
04.04.2021	The number of new coronavirus infections exceeds the **7-day incidence value of 100** for three consecutive days. This means that the **"emergency lockdown"** agreed by the federal and state governments comes into effect in Munich for the first time. This involves stricter contact restrictions and a night-time curfew from 10 pm to 5 am. Cultural and leisure facilities have to close again. Pre-ordered goods may only be collected from local retailers ("Click & Collect").	
12.04.2021	Due to the requirements of the State of Bavaria, **two negative corona tests per week** are now mandatory **for students** to participate in on-campus classes. With a 7-day incidence of over 100, the testing frequency of at least twice a week is mandatory.	
16.04.2021	In order to prevent gatherings of people and prevent infections, the consumption of alcohol at Gärtnerplatz and Wedekindplatz is prohibited daily from 6 p.m. to 6 a.m. with immediate effect. The **ban on alcohol consumption** in the pedestrian zone has been adjusted and applies daily from 6 pm to 6 am. At Viktualienmarkt, alcohol may still not be consumed in public spaces at any time.	
24.04.2021	The City of Munich offers vaccination with AstraZeneca at the ISAR Clinic to 6.000 Munich residents as part of the special **"Vaccinate 60+" campaign**. The first six days of the week-long vaccination campaign are fully booked just a few hours after registration opens.	
03.05.2021	The world's largest fair, the **Munich Oktoberfest**, is once again canceled this year due to the coronavirus pandemic. This was announced by Mayor Dieter Reiter and Bavaria's Minister-President Dr. Markus Söder.	
03.05.2021	The City **expands** its communal **coronavirus test center** at Theresienwiese. Citizens now have the opportunity to get either a free PCR test or a free rapid coronavirus test at this site. In total, up to 2.000 tests per day are possible at the new station. In addition, numerous **private test centers** and pharmacies offer rapid tests for citizens.	
11.05.2021	The incidence classification "50-100" applies again for Munich. This means, among other things, that the night-time curfew will be abolished, contact **restrictions may be eased** and testing will no longer be mandatory in stores for appointment shopping ("Click & Meet").	
20.05.2021	The health department launches its decentralized vaccination services in Munich with **mobile vaccination stations**. Vaccinations are offered in buses at varying locations.	
28.05.2021	The pilot project "**Digital crowd indicator** for particularly busy locations" at Gärtnerplatz goes online. This provides information on which places are already heavily frequented and should therefore be avoided. Gerner Brücke and Wedekindplatz are also included in the digital crowd indicator.	
30.05.2021	Following a further decline in the number of coronavirus cases, the incidence classification "under 50" applies to Munich. This means that all stores are allowed to **reopen** - with a limit on the number of customers - without the obligation to book appointments and re-register. Regular childcare services are possible again. In addition, the city allows further relaxing of the rules with the approval of the Ministry of Health: When visiting the outdoor restaurants (until 10 p.m.), the requirement to test and to book an appointment in advance does not apply. Testing is also no longer mandatory in theaters, outdoor pools and fitness studios.	
02.06.2021	**Contact restrictions** are once again **eased** and based on the incidence classification "under 35". This means that private gatherings are possible for members of one's own household and two other households with a maximum of ten people in total.	
07.06.2021	The **state of emergency** caused by the coronavirus pandemic is **revoked** for the State of Bavaria.	
07.06.2021	The 500.000th coronavirus vaccination is registered at the Riem vaccination center. Another milestone in the vaccination campaign follows four days later: the number of all **vaccinations** performed in Munich exceeds the **one million** mark.	

09.06.2021	According to a decision by the Staff for Extraordinary Events (SAE) under the leadership of Mayor Dieter Reiter, the obligation to wear **face masks outdoors is revoked** in the city center. In addition, the **ban on alcohol consumption** in public spaces is **relaxed.**	
23.06.2021	With immediate effect, there is a **night-time ban on glass containers** in the northern part of Türkenstraße. It is forbidden to carry and use glass bottles, glasses or jugs in public areas of Türkenstraße between Schellingstraße and Akademiestraße and at Georg-Elser-Platz from 8 p.m. to 6 a.m. daily. Two days later, the ban on glass bottles is also introduced at Gärtnerplatz and Wedekindplatz. The **night-time alcohol consumption bans** on Gärtnerplatz and Wedekindplatz can no longer be maintained due to the decreased coronavirus incidence rates and are **dropped**.	
02.07.2021	As for all vaccination centers in Bavaria, **vaccination prioritization is revoked** for the Riem vaccination center. With the change in the state's system, separate proof of priority such as age, occupational group or previous illnesses is no longer required for a vaccination appointment. However, individuals who are part of the previously prioritized groups 1 and 2 will still be given preferential treatment.	
